# Polysubstance Use Patterns among Outpatients Undergoing Substance Use Disorder Treatment: A Latent Class Analysis

**DOI:** 10.3390/ijerph192416759

**Published:** 2022-12-14

**Authors:** Natale Salvatore Bonfiglio, Igor Portoghese, Roberta Renati, Maria Lidia Mascia, Maria Pietronilla Penna

**Affiliations:** 1 Department of Pedagogy, Psychology, Philosophy, University of Cagliari, 09126 Cagliari, Italy; 2 Noah SRL, 27100 Pavia, Italy; 3 Department of Medical Sciences and Public Health, University of Cagliari, 09124 Cagliari, Italy

**Keywords:** addiction, dependence, latent class analysis, polyabuse

## Abstract

Substance Use Disorders (SUDs) pose significant challenges to both individuals and society at large. The primary focus of existing research with clinical SUD populations has been on individual substances, but research is required to better understand the profiles of individuals who use different substances simultaneously. The purpose of the current study was, therefore, to identify patterns of use among subjects (*n* = 1025) who reported using multiple substances by adopting a Latent Class Analysis (LCA) methodology. The Addiction Severity Index (ASI-lite) was included as a measure of substance misuse, we performed LCA to identify patterns of substance use through the administration of the ASI-Lite. Responses were collected from the following substances: alcohol, cannabis/cannabinoids, opioids and heroin, and cocaine. Results identified two latent classes: (1) alcohol use dominant, and (2) poly-abuser use dominants. Class 1 represented 60.0% of the sample and refers to individuals with the dominant use of alcohol, of those a higher proportion (47%) reported low-frequency use (1 to 7 days per month) and 26% reported a frequency of use of 24 to 30 days per month. Furthermore, 18% used alcohol in combination with cocaine. Class 2 represents 40.0% of the sample. This class is characterized by low-frequency and high-frequency users of several substances. The results obtained highlight the importance of deepening the study of the concomitant use of substances in individuals with SUDs to better understand the health risk of the combined use of two or more substances.

## 1. Introduction

The simultaneous (at the same time) or on separate occasions (sequential use) consumption of more than one drug over a given period is broadly defined as polysubstance use [1]. Most of the research on substance use disorders (SUD) has focused on the use of an individual substance [2], giving less importance to polysubstance use engaged by drug users, for example, the simultaneous use of cocaine and heroin or the frequent combined use of cannabis with other illegal substances [3,4,5,6,7,8]. Indeed, considering both sequential and simultaneous polydrug use, the average of substances used at the same time is 3.5, as reported by drug-dependent individuals [2,9]. Despite the variation in the combination of drugs, typically the primary drugs (or dependence) are alcohol, opioids or heroin, and amphetamine/methamphetamine, while cocaine and cannabis are reported to be the secondary-or tertiary substance of use [2].

The rate of multiple drug use disorders has been significantly increasing in recent years [10]. Studies on study prevalence have indicated a high rate of polydrug use among individuals with opioid use disorder (30–49.7%) or in patients receiving treatment (65%) [10]. Moreover, more than two-thirds (79%) of subjects reporting the combination of cocaine and heroin or opioid uses declared to have followed one or more service treatments.

Users report the intentional combination of substances to enhance the effects of intoxication or to alleviate the symptoms of withdrawal [11]. Hence, the identification of distinct patterns of substance use is particularly useful for researchers and clinicians during treatment programs. For example, the use of multiple drugs could be problematic in a treatment setting to maintain methadone therapy [12,13,14]. Furthermore, identifying which substances tend to be used together can help make therapeutic approaches more effective and better understand the risks to physical, mental, or social functioning [15,16,17].

The high rate of polysubstance use is alarming given the impact it can have on both the severity and treatment outcomes of SUDs [2]. Individuals who are polysubstance users are more resistant to change over time, and the unchanged pattern of polysubstance use could foster underlying social or individual problems and risk factors [18].

Polydrug use can be unfavorable to the effectiveness of treatment programs since patients engaging in the use of more drugs simultaneously or concurrently are at increased risk of dropping out [19,20] or less responsive to treatment [21,22,23,24] or more impulsive [25,26].

Polydrug use is also associated with worse outcomes and has a high risk of relapse [27,28] and premature mortality due to drug overdose [29], exhibiting aggressive behavior, suicidal ideation and attempts [30,31]. Yang and colleagues have recently reported an association between polydrug use and major depression, dysthymia, in a study evaluating polydrug use among Chinese heroin users [32].

One of the main limitations of several studies on substance use is to not consider more than two substances as patterns of polysubstance (e.g., alcohol only, marijuana only) [33] or to identify few classes, for example, no/low use, polysubstance use, and single substance classes [34,35]. For this reason, a deeper understanding of polysubstance use as a complex pattern is crucial because of its high intrinsic degree of complexity.

Recently, research using Latent Class Analysis (LCA) focused on identifying patterns of use of multiple substances [21,36,37,38,39,40]. LCA has been used in several studies to empirically detect substance use patterns [41,42,43,44] using observed characteristics properly combined to identify unobserved classes (defined as latent) within a heterogeneous sample [36]. Based on the similarity of response patterns, LCA can also be used to identify subgroups starting from a heterogeneous population [45]. It is possible, therefore, to identify through LCA subtypes of subjects exhibiting similar patterns of characteristics.

As the use of multiple substances among individuals becomes more prevalent, the need to know the patterns of co-occurring substance use become more important to better facilitate the creation of personalized treatment programs [17,24]. We aim, therefore, to employ LCA to discover current (past 30-days) patterns of polysubstance use among subjects who reported substance use in their lifetime. In addition, substance users’ demographic characteristics (e.g., age) have been used to identify high-risk categories of subjects.

## 2. Materials and Methods

### 2.1. Participants

The study was conducted at some outpatient centers in Milan (Italy). Participants were attending government specialist addiction treatment services located in Italy. Subjects entered the treatment pathway after being referred by other services or voluntarily. After the initial referral, subjects were entered on a waiting list. Once vacancies became available, subjects were provided with an initial psychiatric interview and a second psychological interview for diagnostic purposes. They would then fill out the ASI-lite lasting 30 min, which was administered by an outside psychological professional in test administration. Subjects could then be in acute intoxication during this entry phase. Following this initial assessment phase, subjects were taken into treatment.

To be included in the study, patients have to be (i) older than 18 years old, and (ii) diagnosed according to DSM- 5 (diagnostic and statistical manual of mental disorders, fifth revision) (Salvatore Bonfiglio et al., 2021 [28]).

During the study period (January 2015 to December 2020) 2750 individuals completed an assessment at several services for psychoactive substance treatment. The dependence was defined as a disorder in accordance with DSM-5) criteria, with substance misuse lasting more than six months and not following any medical treatment for substance abuse (e.g., methadone). Considering the inclusion and exclusion criteria, this left a final sample of 1754 individuals (269 subjects using methadone and, 727 subjects that did not use any one or more substances within the last 30 days were excluded).

Eligible patients provided written informed consent. The study protocol fully adhered to the guidelines of the ethics committee of the University of Pavia (Italy). Data and information regarding the study were kept confidential from participants and managed in accordance with the relevant provisions (EU Regulation 2016/679-RGDP) and the “code of ethics and good conduct for the processing of personal data for statistical and scientific purposes (provision of the guarantor no. 2 of 16 June 2004)”. Anonymity was guaranteed using codes.

### 2.2. Measures

Demographics, type of substance, and frequency of use in the last 30 days were collected from an admission log compiled when subjects were admitted into the service unit.

The Addiction Severity Index (ASI-lite) was used, to include measures of substance misuse from the following drugs in the previous 30 days: alcohol, cocaine, opioids, heroin, amphetamines, cannabis, other opioids [19,46]. The ASI-lite is a semi-structured interview developed to collect information from patients to identify gravity of psychoactive substances use [47]. The following problem areas are covered by the ASI interview: 1. medical; 2. employment; 3. use of alcohol; 4. use of other substances; 5. legality; 6. family and social functions; 7. Psychiatric. The lite version of the ASI consists of a small number of items (125) compared to the original version. The items selected in the lite version can be quantified; it is then possible, from these, to calculate a composite score in relation to each area. Items were translated into Italian and then back-translated into English by a native English speaker. For this study, the following questions “How many days in the past 30 did you use alcohol/cocaine/heroin/opioid/cannabis (any use at all)?”, and “How many days in the past 30 did you use more than one substance per day?” have been used.

### 2.3. Data Analysis

An LCA was performed in order to identify category patterns of use from ASI-Lite responses relative to the following five substances: alcohol, cannabis or THC or cannabinoids, opioids, cocaine and heroin. Amphetamines/methamphetamines and barbiturates/sedatives were removed from the analyses due to their low rate of use. The ASI-Lite scores were reclassified to reflect the number of times per week of use for each substance. We hypothesized that our data would have a zero-inflated Poisson distribution for each substance, and therefore, we included the following categorical classifications: 0 (0 days of use reported), 1 (1–7 days per month), 2 of use per week (8–15 days per month), 3 (16–23 days per month), and 4 (24–30 days per month).

The robust maximum likelihood (MLR) estimator MPlus 7 was used to estimate LCAs. We have included from one to six classes to estimate. LCAs were conducted using 5000 random sets of values and 1000 iterations to avoid converging [48,49]. We retained classes considering the consistency with theoretical meaning, the conformity of the extracted classes and the statistical appropriateness of the extracted solution [50,51,52].

The following goodness-of-fit indices were measured: the Akaike information criterion (AIC), the Bayesian information criterion (BIC), the Sample Adjusted Bayesian information criterion (SABIC), the approximate Bayes factor (BF), the Constant AIC (CAIC) and the approximate correct model probability (cmP). Smaller AIC, BIC, SABIC and CAIC values indicate a better fit. The BF compares two models at a time (k and k + 1 model) and the k-class model with BF > 3 is considered the best and more parsimonious model. The cmP compares all models under consideration and the model with cmP > 0.10 could be considered a candidate model. Furthermore, two statistical tests were considered: the adjusted Lo–Mendell–Rubin likelihood ratio test (adjusted LMR-LRT; [53]) and the parametric bootstrapped likelihood ratio test (BLRT; [49]). Both tests compare a (k − 1) class model with a k-class model and non-significant *p* values support the k − 1 class model. Moreover, a larger degree of separation between classes is indicated by a higher entropy. Information criteria are visually presented through “elbow plots”, showing the improvements related to additional classes [54]. More precisely, when the slope flattens, an optimal number of classes should be inspected by considering one more and one less class. The assignment of patients to classes was conducted according to their posterior class membership probabilities.

A one-way ANOVA for continuous variables and a Chi-square for qualitative variables were used to evaluate significant differences between the extracted clusters and characteristics of patients.

## 3. Results

### 3.1. Participant Characteristics

A cross-sectional survey study has been carried out. A total of 1354 questionnaires were returned from the initial 1754; missing covariate >5% (400), missing responses >5% (329) and those who declared gambling (120) as their main addiction were removed. The final sample was composed of 905 subjects.

As shown in Table 1, most of the sample subjects (*n* = 841, 82%) were males (mean age = 38.22; SD = 11.73). The mean age of first use of illicit substances was 23.13 (SD = 10.42) and the duration of the substance use was 10.85 years (SD = 10.15). Regarding employment, 53.5% were employed.

### 3.2. Latent Profile Analysis

**Figure 1:**
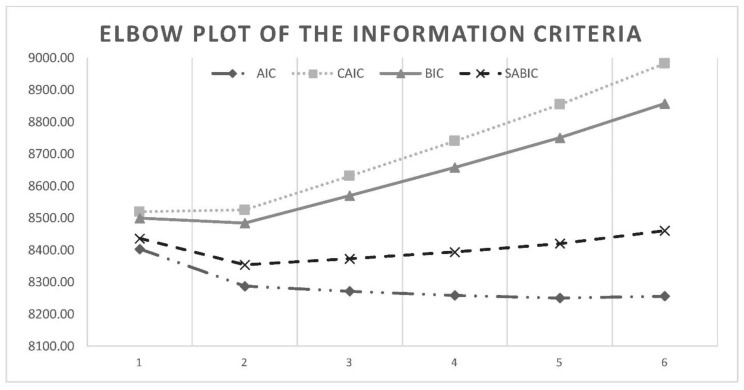
Elbow plot of the information criteria.

LCA was conducted among the 1025 individuals. Table 2 reported the model fit statistics and a brief class description for the solution ranging from 1 to 6-classes. Figure 1 reported the Elbow plot of the information criteria.

Taken as a whole, the two-class solution showed the better fit as it was supported by all the fit indices and BLRT tests (Table 2). The calculated average posterior probabilities of class membership were 0.83 and 0.89, showing low cross-probabilities of 0.17 and 0.11.

Alcohol use dominant (class 1) and polysubstance use dominant (class 2) were the two identified clusters, as shown in Figure 1. The former class represents 60.0% of the sample (*n* = 543, latent class membership probability = 0.82); 47.4% endorsed alcohol use 1 to 7 days per month, and 26.3% endorsed alcohol use more than 24 to 30 days per month. Additionally, nearly everyone did not use heroin and opioids in this group, and only 17.9% of participants used cocaine 1–7 days per month. The second class represents 40.0% of the sample (*n* = 362, latent class membership probability = 0.87). In this class, 24.6% endorsed alcohol use 24 to 30 days per month, and 20.6% endorsed alcohol use 1 to 7 days per month, 11.3% used heroin 24 to 30 days per month and 7.6% used heroin 1 to 7 days per month. 26.9% of participants used cocaine 1 to 7 days per month, and 16.4% used cocaine 16 to 23 days per month; 6.7% used opioids 24 to 30 days per month, and 21.4% used cannabis 24 to 30 days per month.

Table 3 shows the differences between the two types of clusters related to patient characteristics.

A significant difference has been found between cluster type and “main substance” (Χ^2^ = 116; df = 4; *p* ≤ 0.001) and between cluster type and “tobacco use” (Χ^2^ = 17.2; df = 1; *p* ≤ 0.001). Moreover, a significant difference has been found between cluster type and “age” (F_(1904)_ = 40.2; *p* ≤ 0.001), “age first use” (F_(1900)_ = 20.2; *p* ≤ 0.001) and “monthly spending to buy substance” (F_(1882)_ = 4.1; *p* ≤ 0.044).

## 4. Discussion

In this study, we aimed to extend drug addiction research through the identification of poly-abusers’ latent classes among Italian adult drug users attending several addiction treatment services, based on simultaneous substance use. We followed a recent line of research on drug use, in which a person-centered approach is applied, identifying empirical patterns of poly-abusers, as a means of LCA. In our study, we identified two clusters: (1) alcohol use dominant and (2) polyabuser use dominants. The two-class solution appears to be coherent with some of the patterns found in the literature, with a variation in co-used substance combinations, in which alcohol is typically the primary drug of dependence [55,56,57], along with cocaine [58,59], alcohol [60], opioids [27,61,62], or cannabis [63]. For example, the combinations of opioids and stimulants are common with both cocaine (“speedball”) and methamphetamine (“bombita”); the use of opioids to reduce overexcitation following cocaine use and the use of amphetamine or cocaine to prevent opioid-related withdrawal symptoms has been noted [64,65,66]. Cannabis and tobacco are often used simultaneously, especially because tobacco serves as an effective delivery system for cannabis [63].

The first class refers to individuals with a dominant use of alcohol, of those, the higher proportion (47%) reported a low-frequency use (1 to 7 days per month) and 26% reported a frequency of use of 24 to 30 days per month. Furthermore, 18% used alcohol in combination with cocaine. Alcohol is one of the most used drugs, and up to 290 million people have been worldwide diagnosed with alcohol use disorder [67]. Our results are consistent with what has been found in the literature showing that alcohol is frequently used especially with psychostimulants such as cocaine [2,68,69]. A meta-analysis identified that cocaine and alcohol (12% of the population analyzed) were the most common combinations (out of a possible 36 combinations) with a 24–98% range of probabilities for simultaneous use [70] and 37–96% of concurrent cocaine and alcohol use. The greater phenomena of euphoria and increased perception of well-being reported by subjects using cocaine and alcohol may explain the enhanced subjective experience of these drugs. Both human and animal models on post-cocaine anxiety experience, report how alcohol consumption dissipates anxiety that persists after cocaine euphoria [71]. Moreover, studies have shown how combined cocaine and alcohol use can enhance and, respectively, reinforce each other through the properties of each substance. The reinforcing process is addressed via both affective and pharmacokinetic interactions, increasing intake as well as the potential for adverse consequences [70].

Many studies have shown that the psychological consequences associated with the use of alcohol in combination with other drugs are more severe than with alcohol used alone [72,73]. Moreover, also the social consequences have been reported, and particularly related to legal, accidents and health problems [55], lifetime sexually transmitted infections and incarceration [37]. Moreover, co-occurring alcohol disorder and drug use have been associated with a greater frequency of problems associated with the treatment and remission of symptoms [74,75] as well as the prevalence of psychological and social harms [72] and more intense drug consumption and drug-craving [76,77]. Moreover, studies showed that subjects dependent on both alcohol and illegal drug use compared to those with single dependency show comorbidities with mental health problems such as anxiety and depression with a double probability [78]. Compared with subjects with single drug use, those with two or more dependencies and patients receiving treatment for alcohol combined with a second illegal drug are more likely to exhibit affective and antisocial personality disorders [79]. For example, cocaine use in patients who are also alcohol abusers is more likely to result in cocaine-related depression and psychosis compared to users without alcohol abuse [60], or other comorbid mental disorders including anxiety [80,81,82].

The second class refers to poly-abusers who use alcohol in combination with heroin, cocaine, opioids, or cannabis. Class 1 is characterized by low-frequency and high-frequency users for several substances. Some researchers had highlighted poly-abuse patterns [68,83], despite most of the research literature on this topic focusing mainly on the combination of two substances. Even with a moderate rate, the use of alcohol in this category is still present.

Some typical characteristics of the subjects for each of the clusters were highlighted to show a typical profile for each of the clusters. For some of these, some significant differences were found, as presented in Table 3. For example, subjects in cluster 1 use tobacco significantly more, have a higher average age, started using substances at a higher age, and spend significantly less on the substance than subjects in cluster 2. In addition, although not significantly, subjects in cluster 1 are mostly employed, while those in cluster 2 are mostly unemployed, and have about one year more addiction than subjects in cluster 2. Gender is more or less equally distributed in both clusters.

The possibility of highlighting typical profiles with typical characteristics may have important implications in clinical practice to identify appropriate therapeutic interventions and treatments and improve efficacy. It is possible, once it is understood which typical profile or cluster patients fill, to construct ad-hoc interventions based on risk and protective factors. It is possible, moreover, to consider typical characteristics of the subject that adhere to the profile, to tailor an intervention more efficiently.

Alcohol may represent one of the most widely used substances for a poly-abuser. Studies have shown that drinking frequency continues to remain stable also in young and adult people, while the quantity drinking alcohol has been shown to decrease over time, especially binge drinking, [84,85], in relation to demographic characteristics such as age, or the amount of use of other substances [83]. Indeed, considering the motivational models’ perspective [83], alcohol dependence satisfies heterogeneous reasons, such as coping with threats to self-esteem, minimizing negative emotions, self-medication, socializing and so on, in cultural and social contexts in which drugs and other drugs (i.e., alcohol) are accepted or justified by the expectations of individuals in terms of positive outcomes related to substance uses [86,87,88,89].

Overall, the findings of our work support previous works showing that illicit drugs are rarely used in isolation [90,91], and the probability of at least weekly use with adverse interactions with other drugs is high. People often use more than one substance at the same time to produce interactive effects (i.e., synergistic) and/or additive or interactive drug effects [92]; this is why polydrug use poses significant health risks for deleterious outcomes and may be a broad indicator of severity [68]. For example, the combination of alcohol and cocaine produces a unique compound with changes in cocaine metabolism that increase blood cocaine levels, producing the psychoactive metabolite cocaethylene that can enhance the risk of cardiotoxicity or other acute adverse outcomes [60,93]. Among cannabis users, contemporary alcohol consumption may increase the risk for alcohol-related blackouts (i.e., the periods of impaired memory that could form during a drinking episode) [94].

## 5. Conclusions

Polysubstance use could have more serious implications for practice and substance treatment compared to mono-substance use because of its association with worse treatment outcomes, including higher rates of relapse, higher mortality rates and poorer treatment retention. It is necessary to better understand the mechanism that leads individuals with SUDs to polysubstance use, to better understand the health risk of the combined use of two or more substances, including use on the same time (concurrent/simultaneous) or on separate occasions (sequential use). Therefore, understanding how those mechanisms work and what is the impact of polysubstance use on the users is crucial for more successful emergency responses and for improvements in long-term treatment outcomes.

### Limitations and Future Directions

Considering the important strengths of our study, future research should be considered in order to address some limitations. Firstly, the low number of women who participated in the study compared to men should be considered with caution. It should be considered, however, that in Italian treatment centers and services for addiction it is common to find much lower percentages of women in treatment compared to men.

A second limitation concerns the distribution proportion of the participants in relation to their substance of addiction. Some subgroups are unbalanced with respect to this variable, due to a small number of participants. However, this aspect is also linked to the characteristics of the subjects who have access to addiction care services in Italy.

Moreover, we did not measure the percentage of subjects that compiled the ASI-Lite being in an intoxication condition. This could limit the interpretation of the results.

Furthermore, the non-probabilistic sampling procedure should be a problem for the generalization of the results, due to the practical difficulties in collecting data in this population of patients. Finally, as our study was cross-sectional, it remains to be determined whether classes will emerge over time. Future research should investigate the stability of classes over time and the effect of important antecedents.

## Figures and Tables

**Table 1:** Characteristics of the patients.

		*n* (%)	M (SD)
Sex	Male	740 (81.8%)	
	Female	165 (18.2%)	
Age			37.72 (11.61)
Age first use (in years)			22.2 (9.6)
Years of addiction			11 (10.3)
Monthly spending to buy substances (in Euros)			618.5 (1620.6)
Main substance	Alcohol	314 (34.7%)	
	THC/Cannabis	112 (12.4%)	
	Cocaine	361 (39.9%)	
	Heroin	92 (10.2%)	
	Other	26 (2.9%)	
Use Tobacco	Yes	789 (87.2%)	
	No	116 (12.8%)	
Employment status	No	406 (43.51%)	
	Yes	579 (56.49%)	

**Table 2:** Class description and indexes of the latent class analysis.

Model	LL	#fp	Scaling	AIC	CAIC	BIC	SABIC	AWE	Entropy	aLMR	BLRT	BF	cmP
1 Class	−4181.78	20	1.000	8403.55	8519.75	8499.75	8436.24	8529.75		Na	Na	0.46	0.313
2 Classes	−4102.54	41	1.026	8287.08	8525.29	8484.29	8354.08	8545.79	0.489	<0.001	<0.001	71.50	0.678
3 Classes	−4073.73	62	1.060	8271.46	8631.69	8569.69	8372.78	8662.69	0.681	ns	<0.001	81.58	0.009
4 Classes	−4046.24	83	1.043	8258.48	8740.72	8657.72	8394.12	8782.22	0.745	ns	<0.05	102.92	0.000
5 Classes	−4021.07	104	1.078	8250.14	8854.40	8750.40	8420.11	8906.40	0.789	ns	ns	212.30	0.000
6 Classes	−4003.14	125	1.118	8256.29	8982.56	8857.56	8460.57	9045.06	0.827	ns	ns		0.000

Note. AIC = Akaike information criterion; aLMR = adjusted Lo–Mendell–Rubin likelihood ratio test; AWE = Approximate Weight of Evidence; BF = Bayes factor; CAIC = Constant AIC; BIC = Bayesian Information Criterion; cmP = approximate correct model probability; fp = free parameters; LL = log-likelihood; SABIC = Sample adjusted BIC; BLRT = bootstrap likelihood ratio test. Na = not available; ns = non-significant.

**Table 3:** Characteristics of the patients divided into the two different clusters.

		Cluster 1	Cluster 2	*p* Value
Main substance	Alcohol	255 (28.2%)	59 (6.5%)	≤0.001
	Heroin	25 (2.8%)	67 (7.4%)	
	Cocaine	181 (20%)	180 (19.9%)	
	THC/cannabis	64 (7.1%)	48 (5.3%)	
	Other	18 (2%)	8 (0.9%)	
Gender	Male	438 (48.4%)	302 (33.4%)	0.292
	Female	105 (11.6%)	60 (6.6%)	
Employment status	Yes	243 (26.9%)	163 (18%)	0.935
	No	300 (33.1%)	199 (22%)	
Tobacco	Yes	453 (50.1%)	90 (9.9%)	≤0.001
	No	336 (37.1%)	26 (2.9%)	
Age		39.7 (12)	34.8 (10.4)	≤0.001
Age first use (in years)		23.5 (10.7)	20.5 (7.3)	≤0.001
Years of addiction		11.4 (10.1)	10.4 (9)	0.122
Monthly spending to buy substance (in Euros)		529.3 (1878)	754 (1111.1)	0.044

## Data Availability

No data available.
